# Seroepidemiological survey of *Mycoplasma pneumoniae* in children aged 0–6 years in Huzhou city (October–December 2023): a seasonal cross-sectional study

**DOI:** 10.3389/fped.2025.1607910

**Published:** 2025-12-04

**Authors:** Yan Liu, Yuda Wang, Chao Zhang, Xiaofu Luo, Zizhe Zhang, Xiaoqiang Hu, Jianyong Shen

**Affiliations:** Huzhou Center for Disease Control and Prevention (Institute of Health Supervision of Huzhou), Huzhou, Zhejiang, China

**Keywords:** *Mycoplasma pneumoniae*, children, seroepidemiological, immunological, infectious disease

## Abstract

**Objective:**

The study aimed to investigate the seroprevalence and geometric mean concentrations (GMCs) of Anti-*Mycoplasma pneumoniae* immunoglobulin G (Anti-MP-IgG) among children aged 0–6 years in October-December 2023 in Huzhou City, China, and explore the influence of age and gender on seropositivity.

**Design:**

Anti-MP-IgG levels were categorized into negative (<24 AU/mL), borderline (≥24 AU/mL and <36 AU/mL), and positive (≥36 AU/mL) groups. The GMCs and prevalence rates were analyzed according to age and gender. Linear regression and logistic regression analyses were performed to assess trends and associations.

**Results:**

A total of 526 participants were enrolled in the study. The overall GMC of Anti-MP-IgG was 6.21 AU/mL (95% CI: 5.39–7.15). While there was no significant difference in GMCs between genders (*P* = 0.862), a significant increasing trend in GMCs was observed with age (*F* = 16.649, *P* < 0.001). The proportion of positive Anti-MP-IgG levels increased significantly with age, from 10.20% in 0-year group to 32.50% in 6-years group (*P* < 0.001). Logistic regression revealed that age was significantly associated with Anti-MP-IgG positivity (OR: 1.37, 95% CI: 1.21–1.56, *P* < 0.001), while gender was not.

**Conclusions:**

The seroprevalence and GMCs of Anti-MP-IgG showed a clear age-related increasing trend, indicating age as a significant factor for seropositivity in children aged 0–6 years. Further studies are needed to explore underlying mechanisms and the implications for public health strategies.

## 1 Introduction

*Mycoplasma pneumoniae* (*M. pneumoniae)* is one of the leading cause of community-acquired pneumonia (CAP) worldwide, with particularly high incidence and hospitalization rates among children (1, 2). Epidemiological studies estimate that *M. pneumoniae* accounts for approximately 22% of pediatric pneumonia hospitalizations globally, with the highest burden observed in Asia (3). The implementation of non-pharmaceutical interventions (NPIs) during the COVID-19 pandemic (2020–2022) dramatically reduced *M. pneumoniae* transmission (4, 5). However, following the relaxation of NPIs in 2023, a significant resurgence occurred (6, 7), particularly affecting children (8, 9). Recent studies have documented a 2.9-fold increase in childhood infections in Denmark (10) and a detection rate of 38.8% among hospitalized children in Shanghai (11), accompanied by altered seasonal patterns and increased mixed infections.

*M. pneumoniae* infections not only cause acute respiratory illnesses, but may also result in complications such as chronic cough (12), asthma (13), which can adversely affect a child's physical and mental well-being and pose a significant public health concern (14). Immune responses to *M. pneumoniae* exhibit age-dependent maturation, progressing from passive maternal antibody protection in early infancy to the development of robust adaptive immunity after two years of age (15). Following infection, the host typically produces specific IgG antibodies against major immunodominant antigens, and the quantitative changes in these antibody levels serve as important indicators of both infection history (exposure) and host immune competence (protective status) (16). Measuring IgG antibodies in asymptomatic children is particularly valuable for detecting subclinical infections that contribute to transmission, identifying population immunity gaps that may predict outbreak risks, and revealing age-specific vulnerability patterns through antibody dynamics. Currently, epidemiological data on *M. pneumoniae* IgG antibody (Anti-MP-IgG) levels in healthy children remain limited, as most studies had focused exclusively on acutely infected populations (17). This gap is particularly pronounced for age-stratified seroprevalence patterns and gender-specific variations, despite their importance for understanding population immunity dynamics. Therefore, assessing the immunological status of *M. pneumoniae* infections in children is critical for developing effective disease prevention and control strategies. Foundational data on antibody levels in healthy children, particularly across different age groups, remain scarce.

This study collected serum samples from healthy children aged 0–6 years between October and December 2023 to measure Anti-MP-IgG levels. The aim was to elucidate the distribution characteristics of antibody levels in this population and explore potential influencing factors. Through this research, we seek to bridge the gap in foundational data on Anti-MP-IgG levels in children, providing a scientific basis for enhancing disease surveillance and prevention strategies for children.

## 2 Materials and methods

### 2.1 Survey subjects and Serum samples

Our cross-sectional study was conducted from October 1st to December 31st 2023 in Huzhou City, China, which comprises three counties and two districts. A multistage stratified random sampling method was employed to select the participants. Firstly, we randomly selected one county (Deqing County) and one district (Wuxing District) by lottery. Secondly, a street within the chosen county/district was randomly selected, respectively. Thirdly, the study subjects were randomly selected by systematic random sampling from the community health examination population with health status verified by certified doctors within October 1st to December 31st 2023. The participants were sampled across seven age groups: 0-year [<12 months], 1-year [≥12 months and <24 months], 2-years [≥24 months and <36 months], 3-years [≥36 months and <48 months], 4-years [≥48 months and <60 months], 5-years [≥60 months and <72 months], 6-years [≥72 months and <84 months]. The inclusion criteria were no acute/chronic conditions, and no respiratory symptoms within 14 days (confirmed by parental report and clinician confirmation). Exclusion criteria included individuals with respiratory symptoms such as fever, cough, and wheezing in the past two weeks, as well as those with acute or chronic infectious diseases, recent trauma or surgery, malignant tumors, immunodeficiency diseases, liver/kidney dysfunction, and neurological disorders that might impact the results. Based on an existing research, the seropositivity rate of Anti-MP-IgG in children was 45% (18), with *α* = 0.05, 1-*β* = 0.90, and *d* = 0.1*p*, the minimum required sample size was calculated using the standard formula for cross-sectional prevalence studies: $N=\frac{Z_{1-\alpha /2}^{2}\times P(1-P)}{d^{2}}$ (19). The calculated minimum sample size was 467 individuals. Accounting for a 10% loss to follow-up, a minimum of 514 individuals needed to be surveyed. Demographic information such as birthday, gender, age and other relevant factors, along with 2–3 mL of serum specimens, were collected from the study subjects.

### 2.2 Laboratory testing

All samples were stored at −80°C before testing. Anti-MP-IgG was detected by chemiluminescence immunoassay (CLIA) using commercial kits, which has demonstrated high sensitivity and specificity in population (20). The experimental tests were conducted by Hangzhou Edicon Medical Laboratory Co. Ltd, Hangzhou, China (CAP-accredited facility). The test procedure strictly followed the instructions provided with the kit. According to the kit instructions, individuals with an Anti-MP-IgG concentration ≥36 AU/mL were considered of serum positivity. A concentration of Anti-MP-IgG <24 AU/mL was indicative of serum negativity. Anti-MP-IgG concentrations ≥24 AU/mL and <36 AU/mL were considered a gray zone and should be interpreted with caution. The detection range of the kit was ≥2 AU/mL and ≤300 AU/mL.

### 2.3 Statistical analysis

The Anti-MP-IgG concentrations were logarithmically transformed, and the geometric mean concentration (GMC) was calculated. Values beyond the detection limit were included in the statistical analysis using the kit's instructions, with a value of 2 AU/mL assigned for samples registering below the detection threshold and 300 AU/mL for samples over the detection threshold. The analysis involved the calculation of the GMC, serum positivity rate, and negative rate of Anti-MP-IgG across different genders, age groups. Single-factor analysis of variance was employed to compare the inter-group differences in GMC levels, and linear regression was used to test the trendiness of GMC. Inter-group rate comparisons were conducted using the chi-square test or fisher test, and trend analyses utilized the Cochran-Armitage trend test. Logistic regression analysis was used to assess the effect of age and gender on the positive rate of Anti-MP-IgG. All statistical tests were two-sided with a significance level *α* set at 0.05. Statistical analysis was conducted using R 4.2.2 software.

### 2.4 Ethics statement

The study was approved by the human research ethics committee of Huzhou Center for Disease Control and Prevention (Ethics Approval Number: 2025Y001). All participants or their guardians were informed and provided with a written informed consent form.

## 3 Results

### 3.1 Basic characteristic of study population

This study enrolled a total of 526 eligible participants, with 279 males and 247 females, yielding a male-to-female ratio of 1.13:1. Statistical analysis revealed no significant gender difference (*χ*^2^ = 1.947, *P* = 0.163). Detailed demographic characteristics are summarized in Table 1.

**Table 1:** The baseline characteristics of study subjects.

Groups	Study numbers	*χ*^2^ value	*P*
Gender		1.947	0.163
Male	279		
Female	247		
Age (year)		18.646	0.004
0-year	98		
1-year	73		
2-years	51		
3-years	67		
4-years	69		
5-years	88		
6-years	80		
Total	526		

### 3.2 The GMC of anti-MP-lgG

**Figure 1:**
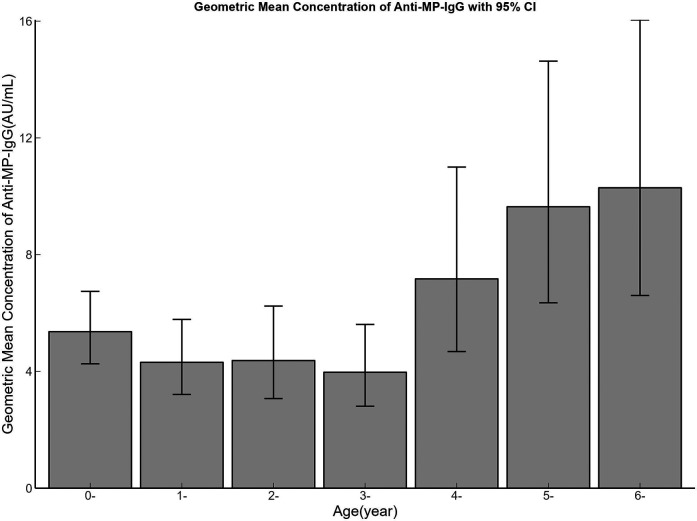
Bar plot of geometric mean concentration of Anti-MP-IgG with 95% CI across different age groups (0–6 years). Geometric mean concentrations (GMC) of Anti-MP-IgG antibodies stratified by age. Bars represent GMC in arbitrary units per milliliter (AU/mL), with vertical lines indicating 95% confidence intervals. Age groups are labeled as “X-” (e.g., “0-” represents infants under 1-year; “1-” represents age ≥1-year and <2-years and so on).

**Figure 2:**
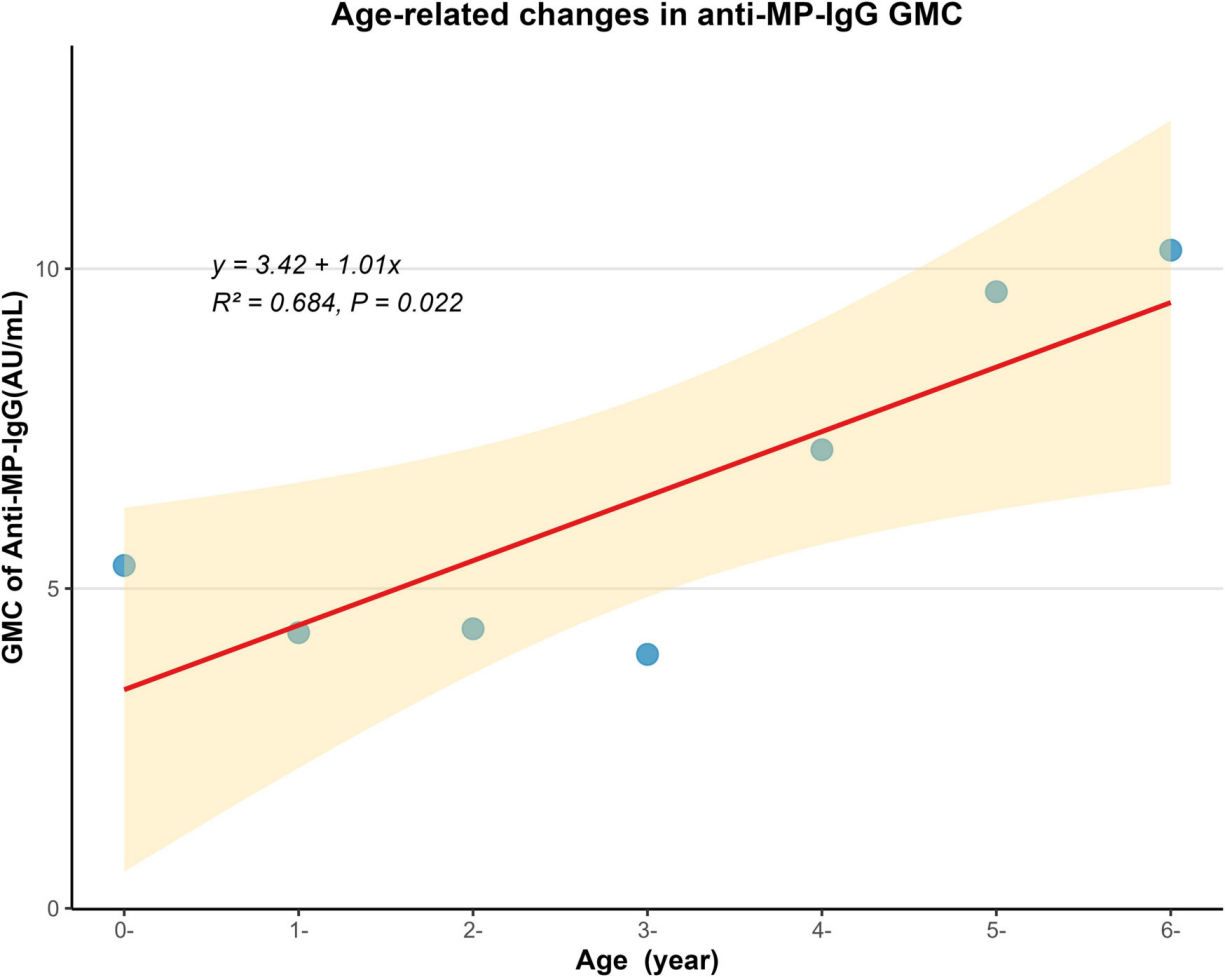
Scatter plot with linear regression: positive correlation between age and Anti-MP-IgG geometric mean concentration (GMC, AU/mL). GMC represents Geometric mean concentrations. Anti-MP-IgG represents anti-*Mycoplasma pneumoniae* IgG antibodies. Age groups are labeled as “X-” (e.g., “0-” represents infants under 1-year; “1-” represents age ≥1-year and <2-years and so on).

The GMC of Anti-MP-lgG for all study subjects was 6.21 (95% CI: 5.39–7.15) AU/mL. The GMC for males was 6.28 (95% CI: 5.17–7.63) AU/mL, and for females, it was 6.13 (95% CI: 4.99–7.53) AU/mL, with no statistically significant difference in gender (*F* = 0.030, *P* = 0.862). While there were statistically significant differences in GMC levels across different age groups (*F* = 16.649, *P* < 0.001). The 6-years group has the highest GMC, which was 10.29 (95% CI: 6.60–16.04) AU/mL. The GMC of 0-year, 1-year, 2-years, 3-yeas, 4-years, 5-years age groups were 5.36 (95%: 4.26–6.74) AU/mL, 4.31 (95%: 3.21–5.78) AU/mL, 4.37 (95%: 3.07–6.24) AU/mL, 3.97 (95%: 2.81–5.61) AU/mL, 7.17 (95%: 4.68–11.00) AU/mL, 9.64 (95%: 6.35–14.63) AU/mL respectively. See Figure 1 for details. Linear regression analysis showed that GMC showed an increasing trend with age (*F* = 10.180, *P* = 0.022, Multiple R-squared = 0.684). See Figure 2 for details.

### 3.3 Prevalence of anti-MP-lgG

Table 2 summarizes the distribution of Anti-MP-IgG levels in children aged 0–6 years of Huzhou City in 2023, stratified by gender and age. Among the total population of 526 children, the majority (81.18%) exhibited negative Anti-MP-IgG levels (<24 AU/mL), 1.14% showed borderline levels (≥24 AU/mL and <36 AU/mL), and 17.68% had positive levels (≥36 AU/mL).

**Table 2:** Distribution of Anti-MP-IgG levels in children aged 0–6 years of Huzhou city in 2023.

Groups	Number	Anti-MP-IgG < 24 AU/mL			24 AU/mL ≤ Anti-MP-IgG <36 AU/mL		Anti-MP-IgG≥36 AU/mL		
		*N* (%)	*χ*^2^ value	*P*	*N* (%)	Fisher *P*	*N* (%)	*χ*^2^ value	*P*
Gender			0.049	0.825		0.689		0.002	0.969
Male	279	225 (80.65)			4 (1.43)		50 (17.92)		
Female	247	202 (81.78)			2 (0.81)		43 (17.41)		
Age (year)			33.719	<0.001		0.806		35.136	<0.001
0-year	98	87 (88.78)			1 (1.02)		10 (10.2)		
1-year	73	67 (91.78)			1 (1.37)		5 (6.85)		
2-years	51	45 (88.24)			1 (1.96)		5 (9.8)		
3-years	67	60 (89.55)			0 (0)		7 (10.45)		
4-years	69	54 (78.26)			1 (1.45)		14 (20.29)		
5-years	88	60 (68.18)			2 (2.27)		26 (29.55)		
6-years	80	54 (67.5)			0 (0)		26 (32.5)		
Total	526	427 (81.18)			6 (1.14)		93 (17.68)		

Gender differences were not statistically significant. For males, 80.65% were negative, 1.43% were borderline, and 17.92% were positive. For females, 81.78% were negative, 0.81% were borderline, and 17.41% were positive. The chi-square tests revealed no significant differences in the proportions of negative (*χ*^2^ = 0.049, *P* = 0.825) or positive (*χ*^2^ = 0.002, *P* = 0.969) cases between genders, and Fisher's exact test for the borderline category was also nonsignificant (*P* = 0.689).

Age stratification revealed significant trends. The proportion of children with negative Anti-MP-IgG levels decreased steadily with age, from 88.78% in the 0-year group to 67.50% in the 6-years group (*χ*^2^ trend *Z* = −0.302, *P* = 0.003). Conversely, positive Anti-MP-IgG levels increased with age, from 10.20% in the 0-year group to 32.50% in the 6-years group (*χ*^2^ trend *Z* = 3.121, *P* < 0.001). Borderline cases were rare and showed no clear trend across age groups. Chi-square tests indicated highly significant differences in the proportions of both negative (*χ*^2^ = 33.719, *P* < 0.001) and positive (*χ*^2^ = 35.136, *P* < 0.001) cases among the different age groups, while Fisher's exact test for the borderline category showed no significant differences (*P* = 0.806).

### 3.4 Logsitic regression analysis

Logistic regression analysis was performed to evaluate the association between Anti-MP-IgG positivity and potential influencing factors, including gender and age (Table 3). The model revealed a significant positive association between age and the likelihood of Anti-MP-IgG positivity. Specifically, the odds ratio (OR) for age was 1.37 (95% CI: 1.21–1.56, *P* < 0.001), indicating that for each additional year of age, the odds of Anti-MP-IgG positivity increased by 37%. Gender, however, was not found to be a significant predictor of Anti-MP-IgG positivity. The OR for gender (female vs. male) was 0.92 (95% CI: 0.64–1.31, *P* = 0.707), suggesting no meaningful difference in positivity rates between males and females.

**Table 3:** The results of logistic regression analysis of age and gender.

Variables	Estimate	Std. error	*Z*-value	*P*-value	OR (95% CI)
Intercept	−2.59	0.28	−9.216	<0.001	0.08 (0.03–0.19)
Gender	−0.09	0.24	−0.376	0.707	0.92 (0.64–1.31)
Age	0.32	0.06	5.197	<0.001	1.37 (1.21–1.56)

## 4 Discussion

Our study provides the systematic evaluation of *M. pneumoniae* seroprevalence among healthy children aged 0–6 years in Huzhou City during the post-pandemic period of winter in 2023. We observed a strong age-dependent increase in both seroprevalence and Anti-MP-IgG levels. Logistic regression confirmed each additional year of age was associated with 37% higher seropositivity odds (OR = 1.37, 1.21–1.56), peaking at 5–6 years.

The age-dependent increase in seroprevalence aligns with previous studies (5, 21, 22). Specifically, the steepest rise occurred in children aged 4 years to 6 years (20.29% to 32.50%), coinciding with entry into kindergarten environments—a well-documented risk factor for respiratory pathogen transmission due to increased close-contact interactions (22). Recent studies indicate that high rates of healthy children carry *M. pneumoniae* in the upper respiratory tract and that current diagnostic PCR or serology cannot discriminate between *M. pneumoniae* infection and carriage (23), which were important source of infection. *M. pneumoniae* outbreaks commonly occur in environments with close contact, such as schools, universities, institutions, camps, and military bases (24). Generally, the start of the school year is accompanied by an increase in the incidence of *M. pneumoniae* infection (24). While NPIs limited the transmission of SARS-CoV-2, they also reduced the spread of other pathogens such as *M. pneumoniae* during and after lockdown periods. However *M. pneumoniae* has its own epidemic cycle, where a substantial surge in the number of infections occurs approximately every 1–4 years (25).The spread of the pathogen such as *M. pneumoniae* was only delayed, and the population was in a state of low infection for a long time, resulting in a large immune debt. Widespread outbreak of *M. pneumoniae* in the fall and winter of 2023 in many places, especially in the preschool children after children's re-engagement in social activities (11). Studies have showed high incidence of *M. pneumoniae* pneumonia in the second half of 2023 in China (26) and other countries (6, 27), indicating that NPIs prevention and control measures of the COVID-19 epidemic may have only delayed the arrival of the peak of *M. pneumoniae* (7). Our findings confirm that age is a critical determinant of *M. pneumoniae* infection among children aged 0–6 years.

While Anti-MP-IgG levels increased with age, our analysis revealed no significant gender differences in either Anti-MP-IgG levels (*P* = 0.862) or seropositivity rates (*P* = 0.969)—a finding consistent with previous IgG-based studies (28, 29), which probably indicate that biological sex differences may play a minimal role in *M. pneumoniae* transmission dynamics during 0–6 years children (30). This contrasts with reports of gender disparities in acute infections detected via IgM assays (31), a discrepancy likely reflecting fundamental differences in what these antibodies represent. IgM indicates recent acute infection, where reported gender differences may stem from hormonal modulation of acute-phase immune responses or healthcare behavior biases. IgG indicates prior exposure to the pathogen, but cannot distinguish between recent and remote infections. The high seroprevalence of Anti-MP-IgG antibodies among healthy children suggests widespread environmental circulation of the pathogen, efficient transmission routes, and a substantial reservoir of asymptomatic carriers. Epidemiological studies estimate that *M. pneumoniae* accounts for approximately 22% of pediatric pneumonia hospitalizations globally, with the highest burden observed in Asia (3). These findings underscore the importance of accelerating vaccine development to mitigate *M. pneumoniae* infection and reduce the associated burden of CAP.

These findings have important public health and clinical implications. Firstly, the increasing seropositivity rates with age highlight the need for enhanced respiratory disease surveillance among children aged 5–6 years, especially in preschool settings, where close-contact transmission is amplified. School-entry health screenings could integrate *M. pneumonia* serology to identify high-risk cohorts (32). Secondly, while no licensed *M. pneumoniae* vaccine currently exists (33), our results suggest that vaccine development could significantly reduce the burden of infection and associated complications. our finding of age-dependent seroconversion (peaking at 5–6 years) identifies a prime target population for emerging candidates. Moreover, combining antibody testing with clinical symptoms may improve the accuracy of diagnosing *M. pneumoniae* infections, particularly in children. Early and precise diagnosis can help reduce unnecessary antibiotic use and improve treatment outcomes (34).

This study was conducted in Huzhou from October to December 2023, following the relaxation of COVID-19 NPIs and the resumption of children's normal social activities—contexts crucial for interpreting *M. pneumoniae* serological patterns. First, SARS-CoV-2 evolution, particularly Omicron's enhanced transmissibility and reduced pathogenicity, facilitated crowd gatherings (e.g., kindergarten attendance), creating optimal conditions for *M. pneumoniae*'s close-contact transmission and contributing to its resurgence in 0–6 years children (35), which aligns with our finding of a sharp age-related rise in Anti-MP-IgG seropositivity, peaking at 32.50% in 6-years. Second, while COVID-19 vaccines do not target *M. pneumoniae*, they may indirectly modulate host immune responses by reshaping respiratory mucosal immunity and systemic homeostasis, potentially altering IgG production efficiency post-exposure (36)—a possible factor for the higher seropositivity in children aged 4–6 years with high vaccine coverage. Thirdly, overlapping complications of *M. pneumoniae* infection (e.g., chronic cough) and long COVID symptoms pose differential diagnosis challenges (37, 38), which emerging non-invasive tools (breath metabolomics, chest ultrasound, exhaled nitric oxide testing) can address (39–42). These pandemic-related factors contextualize our findings and inform optimized *M. pneumoniae* surveillance and diagnosis in children.

Despite the robust stratified random sampling design of this study, several limitations should be noted. Firstly, the cross-sectional nature of the study precludes causal inferences. Secondly, the geographical restriction to Huzhou City and temporal limitation to winter months (October–December 2023) may affect the generalizability of our findings, as seroprevalence patterns could vary across regions and demonstrate seasonal fluctuations. Thirdly, while we controlled for baseline health status through medical verification, unmeasured environmental exposures (e.g., daycare attendance) may contribute to residual confounding. Prospective designs should incorporate these variables. Additionally, IgG seropositivity should not be interpreted as definitive evidence of protective immunity, and the lack of a unified standard for defining seropositivity thresholds for *M. pneumoniae* antibodies complicates comparisons across studies, limiting direct comparability of our results with those from other researches.

Future studies should consider longitudinal cohort designs to better assess dynamic changes in Anti-MP-IgG levels over time. Further research on the correlation between antibody levels and clinical severity of *M. pneumoniae* infections would also enhance the utility of serological testing in patient management.

## 5 Conclusions

This study provides valuable baseline data on *M. pneumoniae* antibody levels among children aged 0–6 years in winter in Huzhou City, revealing a significant age-related increase in both GMC and seropositivity rates. Age was confirmed as a key determinant of infection exposure and immune response. These findings offer a scientific basis for future public health strategies and underscore the importance of vaccine development and early intervention measures.

## Data Availability

The raw data supporting the conclusions of this article will be made available by the authors, without undue reservation.

## References

[1] Jain S Williams DJ Arnold SR Ampofo K Bramley AM Reed C Community-acquired pneumonia requiring hospitalization among US children. N Engl J Med. (2015) 372:835–45. 10.1056/NEJMoa1405870 25714161 PMC4697461

[2] Xie X-y Zhou R-y Ding S-a Ma B-x Zhang X Zhang Y. Emerging trends and concerns in *Mycoplasma pneumoniae* pneumonia among Chinese pediatric population. Pediatr Res. (2024) 95:1–3. 10.1038/s41390-024-03049-y 38273116

[3] Song Z Jia G Luo G Han C Zhang B Wang X. Global research trends of *Mycoplasma pneumoniae* pneumonia in children: a bibliometric analysis. Front Pediatr. (2023) 11:1306234. 10.3389/fped.2023.1306234 38078315 PMC10704248

[4] Sauteur PMM Beeton ML Pereyre S Bébéar C Gardette M Hénin N *Mycoplasma pneumoniae*: gone forever? Lancet Microbe. (2023) 4:e763. 10.1016/S2666-5247(23)00182-9 37393927

[5] Zhang Y Huang Y Ai T Luo J Liu H. Effect of COVID-19 on childhood *Mycoplasma pneumoniae* infection in Chengdu, China. BMC Pediatr. (2021) 21:202. 10.1186/s12887-021-02679-z 33910509 PMC8079841

[6] Upadhyay P Singh V. *Mycoplasma pneumoniae* outbreak in 2023: post-pandemic resurgence of an atypical bacterial pathogen. Cureus. (2024) 16:e58757. 10.7759/cureus.58757 38779270 PMC11111095

[7] Sauteur PMM Beeton ML Pereyre S Bébéar C Gardette M Hénin N *Mycoplasma pneumoniae*: delayed re-emergence after COVID-19 pandemic restrictions. Lancet Microbe. (2024) 5:e100–101. 10.1016/S2666-5247(23)00344-0 38008103

[8] Liu T-H Wu J-Y Huang P-Y Tsai Y-W Lai C-C. The effect of nirmatrelvir plus ritonavir on the long-term risk of epilepsy and seizure following COVID-19: a retrospective cohort study including 91,528 patients. J Infect. (2023) 86:256–308. 10.1016/j.jinf.2023.01.014 PMC9840225 36649824

[9] Sauteur PMM Beeton ML Uldum SA Bossuyt N Vermeulen M Loens K *Mycoplasma pneumoniae* detections before and during the COVID-19 pandemic: results of a global survey, 2017 to 2021. Euro Surveill. (2022) 27:2100746. 10.2807/1560-7917.ES.2022.27.19.2100746 35551702 PMC9101966

[10] Dungu KHS Holm M Hartling U Jensen LH Nielsen AB Schmidt LS *Mycoplasma pneumoniae* incidence, phenotype, and severity in children and adolescents in Denmark before, during, and after the COVID-19 pandemic: a nationwide multicentre population-based cohort study. Lancet Reg Health Eur. (2024) 47:101103. 10.1016/j.lanepe.2024.101103 39469091 PMC11513821

[11] Liu P Xu M Lu L Zhu X Jia R Dong N Resurgence of common respiratory viruses and *Mycoplasma pneumoniae* after ending the zero-COVID policy in Shanghai. Sci Rep. (2025) 15:1765. 10.1038/s41598-025-85852-z 39800785 PMC11725580

[12] Zheng K Tang L Wang X Chen L Zhao Y Chen X. The risk factors for chronic cough in children: a meta-analysis covering five continents. Respir Med. (2024) 232:107752. 10.1016/j.rmed.2024.107752 39094792

[13] Liu X Wang Y Chen C Liu K. *Mycoplasma pneumoniae* infection and risk of childhood asthma: a systematic review and meta-analysis. Microb Pathogen. (2021) 155:104893. 10.1016/j.micpath.2021.104893 33932544

[14] Xu Y Yang C Sun P Zeng F Wang Q Wu J Epidemic features and megagenomic analysis of childhood *Mycoplasma Pneumoniae* post COVID-19 pandemic: a 6-year study in southern China. Emerg Microbes Infect. (2024) 13:2353298. 10.1080/22221751.2024.2353298 38721691 PMC11212572

[15] Albrecht M Arck PC. Vertically transferred immunity in neonates: mothers, mechanisms and mediators. Front Immunol. (2020) 11:555. 10.3389/fimmu.2020.00555 32296443 PMC7136470

[16] Lee W-J Huang E-Y Tsai C-M Kuo K-C Huang Y-C Hsieh K-S Role of serum *Mycoplasma pneumoniae* IgA, IgM, and IgG in the diagnosis of *Mycoplasma pneumoniae*-related pneumonia in school-age children and adolescents. Clin Vaccine Immunol. (2017) 24:e00471–16. 10.1128/CVI.00471-16 27760779 PMC5216438

[17] Wang X Li M Luo M Luo Q Kang L Xie H *Mycoplasma pneumoniae* triggers pneumonia epidemic in autumn and winter in Beijing: a multicentre, population-based epidemiological study between 2015 and 2020. Emerg Microbes Infect. (2022) 11:1508–17. 10.1080/22221751.2022.2078228 35582916 PMC9176688

[18] Du D Liao S Wu Y Jiao Y Wu D Wu W Serological analysis and drug resistance of chlamydia pneumoniae and *Mycoplasma pneumoniae* in 4500 healthy subjects in Shenzhen, China. Biomed Res Int. (2017) 2017:3120138. 10.1155/2017/3120138 29057257 PMC5625799

[19] Von Elm E Altman DG Egger M Pocock SJ Gøtzsche PC Vandenbroucke JP. The strengthening the reporting of observational studies in epidemiology (STROBE) statement: guidelines for reporting observational studies. Lancet. (2007) 370:1453–7. 10.1016/j.jclinepi.2007.11.008 18064739

[20] He S Yang M Wu X Cai G Jiang K Xie L. Comparison of a novel chemiluminescence immunoassay with the passive agglutination method for the diagnosis of *Mycoplasma pneumoniae* infection in children. J Microbiol Methods. (2020) 173:105921. 10.1016/j.mimet.2020.105921 32320711

[21] Meyer Sauteur PM Unger WWJ Nadal D Berger C Vink C van Rossum AMC. Infection with and carriage of *Mycoplasma pneumoniae* in children. Front Microbiol. (2016) 7:329. 10.3389/fmicb.2016.00329 27047456 PMC4803743

[22] Kutty PK Jain S Taylor TH Bramley AM Diaz MH Ampofo K *Mycoplasma pneumoniae* among children hospitalized with community-acquired pneumonia. Clin Infect Dis. (2019) 68:5–12. 10.1093/cid/ciy419 29788037 PMC6552676

[23] Meyer Sauteur PM Unger WW Nadal D Berger C Vink C van Rossum AM. Infection with and carriage of *Mycoplasma pneumoniae* in children. Front Microbiol. (2016) 7:329. 10.3389/fmicb.2016.00329. 27047456 PMC4803743

[24] Yan C Xue G-H Zhao H-Q Feng Y-L Cui J-H Yuan J. Current status of *Mycoplasma pneumoniae* infection in China. World J Pediatr. (2024) 20:1–4. 10.1007/s12519-023-00783-x 38185707 PMC10827902

[25] Rowlands RS Meyer Sauteur PM Beeton ML. On behalf of the escmid study group for M, Chlamydia infections E. *Mycoplasma pneumoniae*: not a typical respiratory pathogen. J Med Microbiol. (2024) 73:001910. 10.1099/jmm.0.001910 39475213 PMC11523975

[26] Wu Q Pan X Han D Ma Z Zhang H. New insights into the epidemiological characteristics of *Mycoplasma pneumoniae* infection before and after the COVID-19 pandemic. Microorganisms. (2024) 12:2019. 10.3390/microorganisms12102019 39458327 PMC11509874

[27] Nordholm AC Søborg B Jokelainen P Lauenborg Møller K Flink Sørensen L Grove Krause T *Mycoplasma pneumoniae* epidemic in Denmark, October to December, 2023. Euro Surveill. (2024) 29:2300707. 10.2807/1560-7917.Es.2024.29.2.2300707 38214084 PMC10785206

[28] Liu M Meng K Jiang J Zhang L Sun S. Comparison of serodiagnosis methods for community-acquired *Mycoplasma pneumoniae* respiratory tract infections in children. Medicine (Baltimore). (2023) 102:e34133. 10.1097/md.0000000000034133 37478238 PMC10662900

[29] Jain S Self WH Wunderink RG Fakhran S Balk R Bramley AM Community-Acquired pneumonia requiring hospitalization among U.S. Adults. N Engl J Med. (2015) 373:415–27. 10.1056/NEJMoa1500245 26172429 PMC4728150

[30] Dumke R Jacobs E. Antibody response to *Mycoplasma pneumoniae*: protection of host and influence on outbreaks? Front Microbiol. (2016) 26:39. 10.3389/fmicb.2016.00039 PMC4726802 26858711

[31] Cheng Y Cheng Y Dai S Hou D Ge M Zhang Y The prevalence of *Mycoplasma pneumoniae* among children in Beijing before and during the COVID-19 pandemic. Front Cell Infect Microbiol. (2022) 12:854505. 10.3389/fcimb.2022.854505 35573799 PMC9103471

[32] Li ZJ Zhang HY Ren LL Lu QB Ren X Zhang CH Etiological and epidemiological features of acute respiratory infections in China. Nat Commun. (2021) 12:5026. 10.1038/s41467-021-25120-6 34408158 PMC8373954

[33] Jiang Z Li S Zhu C Zhou R Leung PHM. *Mycoplasma pneumoniae* infections: pathogenesis and vaccine development. Pathogens. (2021) 10:119. 10.3390/pathogens10020119 33503845 PMC7911756

[34] Ding G Zhang X Vinturache A van Rossum AMC Yin Y Zhang Y. Challenges in the treatment of pediatric *Mycoplasma pneumoniae* pneumonia. Eur J Pediatr. (2024) 183:3001–11. 10.1007/s00431-024-05519-1 38634891

[35] Hyug Choi J Sook Jun M Yong Jeon J Kim HS Kyung Kim Y Ho Jeon C Global lineage evolution pattern of sars-cov-2 in Africa, America, Europe, and Asia: a comparative analysis of variant clusters and their relevance across continents. J Transl Int Med. (2023) 11:410–22. 10.2478/jtim-2023-0118 38130632 PMC10732492

[36] Xie J Ye F Deng X Tang Y Liang JY Huang X Circular RNA: a promising new star of vaccine. J Transl Int Med. (2023) 11:372–81. 10.2478/jtim-2023-0122 38130633 PMC10732498

[37] Guo M Shang S Li M Cai G Li P Chen X Understanding autoimmune response after SARS-CoV-2 infection and the pathogenesis/mechanisms of long COVID. Med Rev. (2024) 4:367–83. 10.1515/mr-2024-0013 PMC11495526 39444797

[38] Ewing AG Salamon S Pretorius E Joffe D Fox G Bilodeau S Review of organ damage from COVID and long COVID: a disease with a spectrum of pathology. Med Rev. (2025) 5:66–75. 10.1515/mr-2024-0030 PMC11834749 39974559

[39] Kang S Zheng R. Distribution of the causes of fever of unknown origin in China, 2013–2022. J Transl Int Med. (2024) 12:299–307. 10.2478/jtim-2024-0008 39081273 PMC11284625

[40] Ariyasingha NM Chowdhury MRH Samoilenko A Salnikov OG Chukanov NV Kovtunova LM Toward lung ventilation imaging using hyperpolarized diethyl ether gas contrast agent. Chemistry. (2024) 30:e202304071. 10.1002/chem.202304071 38381807 PMC11065616

[41] Ariyasingha NM Samoilenko A Chowdhury MRH Nantogma S Oladun C Birchall JR Developing hyperpolarized butane gas for ventilation lung imaging. Chem Biomed Imaging. (2024) 2:698–710. 10.1021/cbmi.4c00041 39483636 PMC11523004

[42] Chowdhury MRH Oladun C Ariyasingha NM Samoilenko A Bawardi T Burueva DB Rapid lung ventilation MRI using parahydrogen-induced polarization of propane gas. Analyst. (2024) 149:5832–42. 10.1039/d4an01029a 39530397 PMC11563306

